# Fracture Property of Y-Shaped Cracks of Brittle Materials under Compression

**DOI:** 10.1155/2014/192978

**Published:** 2014-06-09

**Authors:** Xiaoyan Zhang, Zheming Zhu, Hongjie Liu

**Affiliations:** Department of Engineering Mechanics, Sichuan University, Chengdu 610065, China

## Abstract

In order to investigate the properties of Y-shaped cracks of brittle materials under compression, compression tests by using square cement mortar specimens with Y-shaped crack were conducted. A true triaxial loading device was applied in the tests, and the major principle stresses or the critical stresses were measured. The results show that as the branch angle *θ* between the branch crack and the stem crack is 75°, the cracked specimen has the lowest strength. In order to explain the test results, numerical models of Y-shaped cracks by using ABAQUS code were established, and the J-integral method was applied in calculating crack tip stress intensity factor (SIF). The results show that when the branch angle *θ* increases, the SIF *K*_I_ of the branch crack increases from negative to positive and the absolute value *K*_II_ of the branch crack first increases, and as *θ* is 50°, it is the maximum, and then it decreases. Finally, in order to further investigate the stress distribution around Y-shaped cracks, photoelastic tests were conducted, and the test results generally agree with the compressive test results.

## 1. Introduction

Cracks are frequently encountered in many engineering structures, such as rock and concrete, and such cracks usually play a dominative role in structure stability. In order to predict and prevent engineering disasters induced by such cracks, it is necessary to investigate the properties of crack propagation so as to obtain a better understanding of the dominant parameters that control material fracturing. Therefore, a great deal of efforts from theoretical and experimental points of view has been devoted to the study of the physics of crack fracture, and accordingly many significant results have been presented in the literatures [1–5].

Cracks are not always straight, and they may have many different shapes, such as curved cracks, intersected cracks, and branched cracks. Y-shaped crack is one kind of branched cracks and exists widely in structures because they are easily developed as two cracks intersect or coalesce. For the problem of multiple interacting cracks in an infinite plate, Yavuz et al. [6] formulated the stress intensity factor (SIF) by using integral equations expressed in terms of unknown edge dislocation along crack lines, and finally some new and challenging crack interaction problems including branched Y-cracks, two-kinked V-cracks were analyzed. For symmetry Y-shaped cracks under compression, Li and Zhu [7] reported that there is a particular relation between the crack angle and the SIF, and the results showed that the SIF at crack tips is strongly dependent both on crack angle and crack length. By using finite element method, Isida and Noguchi [8] solved the basic problems of branched cracks in an infinite body through diverse numerical methods. Moreover, also by using finite element method, several researchers [9–15] calculated the growing path and the SIF values of branched cracks. For nonstraight cracks, Tilbrook and Hoffman [16] employed a simple analytical model to predict mechanical energy release rate and deflection angle for a range of crack shapes under mixed-mode loading. It was found that the crack length and orientation of the crack tip with respect to loading direction are the key influences on fracture parameters. The experimental study of the coalescence property of two cracks under compression conducted by Bobet and Einstein [17] showed that as the vertical load increases, new cracks emanate from the flaws and eventually coalesce; flaw slippage, wing-crack initiation, secondary crack initiation, crack coalescence, and failure were observed. Two types of cracks arised: wing-cracks, which are tensile cracks, and secondary cracks which initiate as shear cracks in a plane roughly coplanar with the flaw.

In order to investigate the fracture behavior of Y-shaped cracks, experimental and numerical studies are implemented in this paper. The experimental studies include true triaxial compression test and photoelastic test. A true triaxial loading device is applied in the measurement of the compressive critical stress of the cement mortar specimens with Y-shaped cracks, and meanwhile in order to qualitatively investigate the crack tip SIFs for the specimens with various branch angles, photoelastic tests are conducted. In the numerical simulation, the finite element code ABAQUS is applied in the calculation of SIF values of Y-shaped crack tips.

## 2. Compression Tests

**Figure 1:**
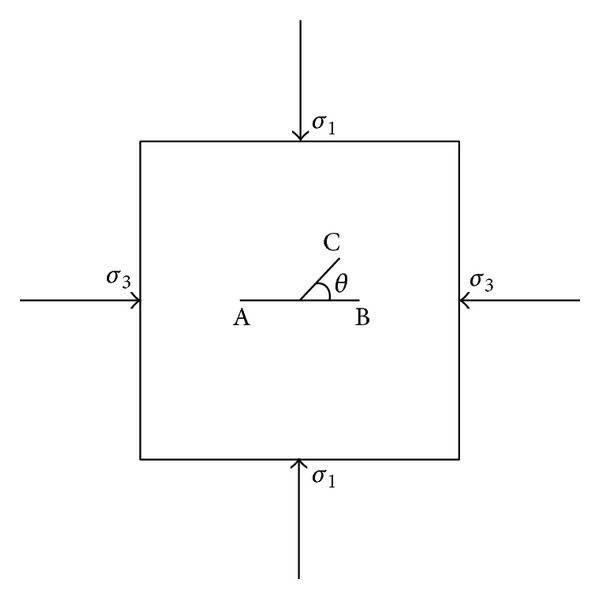
Y-shaped crack under compression.

The specimens were square plates, 150 mm ∗ 150 mm ∗ 50 mm, with a Y-shaped artificial and penetrated crack, as shown in Figure 1. The main crack AB measures 40 mm in length, while the branch crack measures 20 mm, and the branch angle *θ* varies from 15° to 90°. The material is cement mortar, and the ratio of cement : water : sand is 1 : 0.5 : 3 by weight. The cracks were made by using a very thin, 0.1 mm, film. The films were placed inside the samples during the process of casting in a mold until they were loaded. The curing period of the samples is 28 days. It is found that after the specimens have been stored in a heating apparatus with a controlled temperature for more than 2 hrs, the films can be easily pulled out from the specimens.

**Figure 2:**
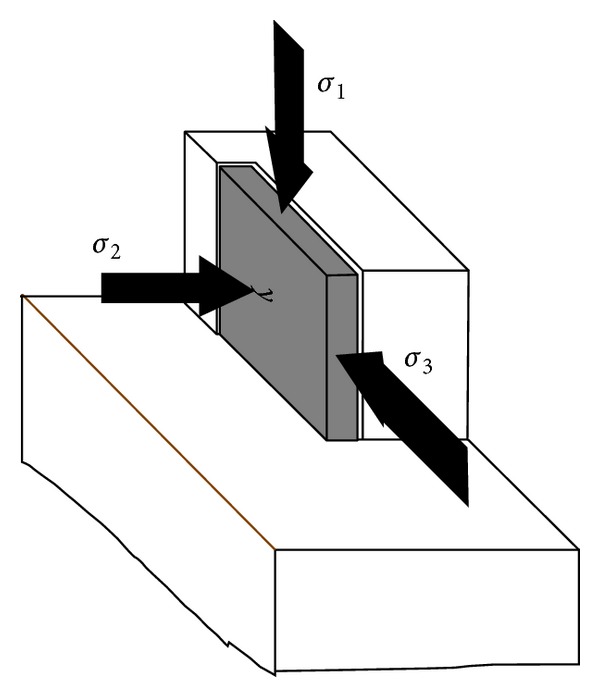
Sketch of a true triaxial loading device.

The specimens were loaded by a true triaxial loading device, as shown in Figure 2. The vertical loading is the major principal stress *σ*_1_, and the two horizontal confining stresses are kept as constants during the process of vertical loading. One of the horizontal stress is *σ*_3_, and the other one is *σ*_2_, In order to avoid the specimen damage before testing, the specimens, at beginning, are loaded simultaneously along three directions, and after *σ*_2_ and *σ*_3_ are fixed to the values predesigned, we continue to increase *σ*_1_ gradually until the specimen fails. The maximum vertical stress *σ*_1_ is selected as the critical stress of the specimens. In order to avoid the effect of the friction between the specimen and the loading device, the specimen surfaces were smeared with oil before testing.

**Figure 3:**
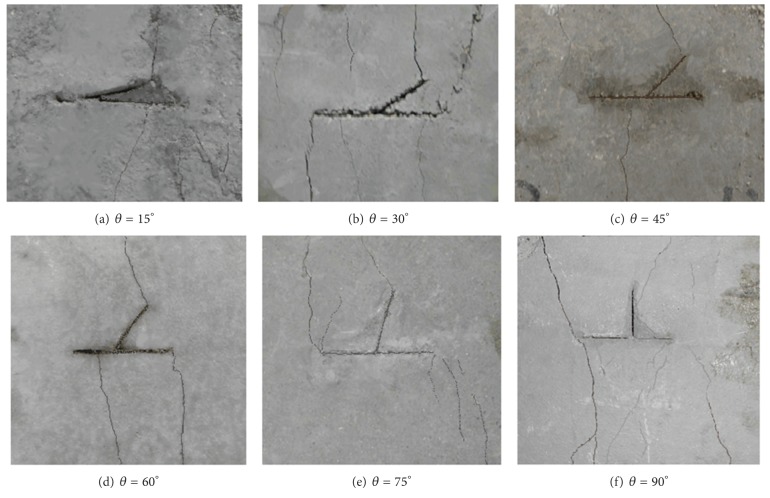
Test result of the fracture patterns for the specimens with different branch angles.

Figure 3 shows the fracture patterns for the specimens with branch angles 15°, 30°, 45°, 60°, 75°, and 90°. It can be seen that wing-cracks often initiate at branch crack tip C and then they grow and curve to the uploading boundary of *σ*_1_.

**Figure 4:**
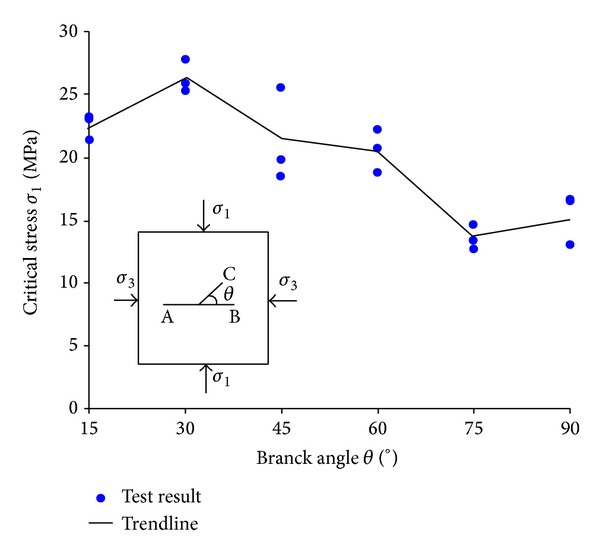
Test result for the relation between the critical stress and branch crack angles *θ*.

Figure 4 shows the test result of the critical stress *σ*_1_ versus branch angle *θ*. As the branch angle is 30°, the average test result of the critical stress is the largest, and as it increases from 30° to 90°, the critical stress generally decreases, and as the branch angle is 75°, the average test result is the lowest.

## 3. Numerical Study

**Figure 5:**
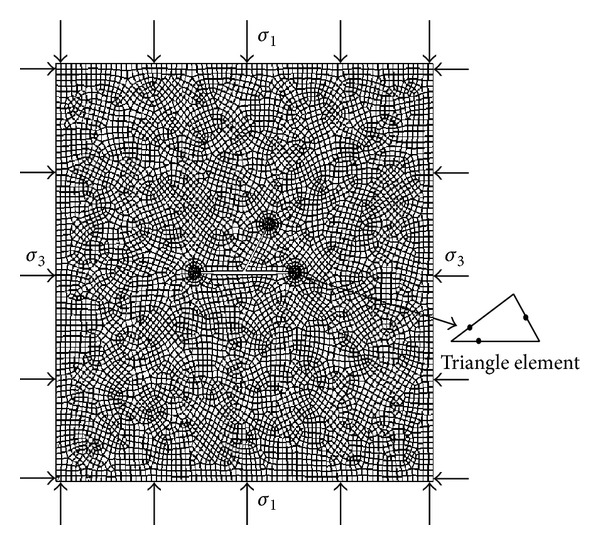
Elements applied in dividing the domain containing Y-shaped cracks.

**Figure 6:**
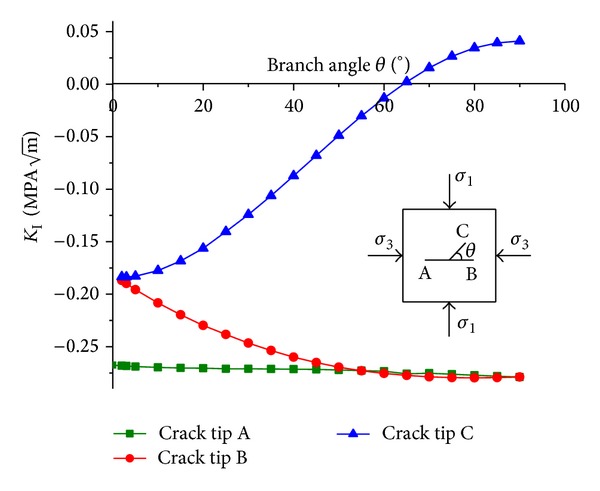
Curves of SIF *K*_I_ versus the branch angle *θ*.

**Figure 7:**
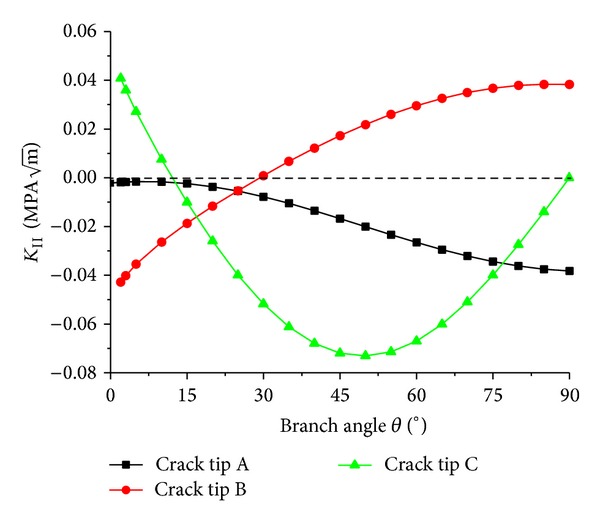
Curves of SIF *K*_II_ versus the branch angle *θ*.

In order to obtain the stress intensity factors of asymmetric Y-shaped cracks, the corresponding numerical study by using ABAQUS code was implemented. In this simulation, except for the zone near crack tips where triangular elements CPS6 were applied, quadrilateral elements CPS8 were employed, as shown in Figure 5. The J-integral or contour integral method was applied in calculating crack tip stress intensity factor (SIF), and the material parameters applied in this numerical study are as follows. Young's modulus is 29.5 GPa; Poisson's ratio is 0.2; the major principal stress *σ*_1_ is 1.0 MPa; and *σ*_3_ is 0.05 MPa. In Total 20 models with different branch angles were established, and the corresponding calculation results are plotted in Figures 6 and 7. For the branch angle *θ* is 15°, 30°, 45°, 60°, 75°, and 90°; the corresponding calculation results of SIF values at crack tips A, B, and C are presented in Table 1.

It can be seen that the values of *K*_I_ at crack tips A and B are always negative. This is because under compression, there is no stress concentration at crack tips, and accordingly the mode I SIF is negative which cannot cause crack propagation. The values of *K*_I_ at crack tip C increase as the branch angles *θ* increase, and as *θ* is larger than 65°, the *K*_I_ changes from negative to positive which could cause crack propagation.

The mode II SIFs *K*_II_ changes with the branch angle *θ*, and the absolute value of *K*_II_ at crack tip A increases slightly with the branch angle *θ*. This is due to the effect from tip C which increases as *θ* increases. As *θ* = 90°, the absolute values of *K*_II_ at crack tips A and B are roughly equal, but their signs are inverse. The absolute value of *K*_II_ at crack tip C increases as *θ* increases from 0° to 50°, and as *θ* = 50°, the *K*_I_ absolute value is the maximum and then it starts to decrease as *θ* increases from 50° to 90°.

From Figures 5 and 6, one can find that for the branch crack C, as *θ* is larger than 65°, the values of *K*_I_ is positive, and meanwhile the absolute values of *K*_II_ is large. Combing the values of *K*_I_ and *K*_II_, one can find that as the branch angle *θ* is in the range between 70° and 75°, the compressive strength of the cracked sample should be low, which agrees with the test results shown in Figure 4.

## 4. Photoelastic Tests

Photoelasticity is an experimental technique for stress and strain analysis that is particularly useful for the samples with a complex geometry and loading conditions. It is a nondestructive, whole-field, graphic stress-analysis technique based on an optical mechanical property called birefringence [18, 19].

According to the standard of marking photoelastic specimen method, a group of specimens as shown in Figure 1 with the inclined angles 0°, 15°, 30°, 45°, 60°, 75°, and 90° are conducted, respectively. The material was polycarbonate (PC) which possesses high transparency. The size of the specimen was 100 mm ∗ 100 mm ∗ 8 mm, and the width of cracks in the specimens was 1 mm.

According to stress-optical law of plane photoelastic experiment, the maximum shear stress *τ*_*m*_ can be calculated based on the following equation:

(1) $$\begin{array}{c} \tau_{m}=\frac{1}{2}\left(\sigma_{1}-\sigma_{3}\right)=\frac{Nf_{\sigma }}{2h}, \end{array}$$

where *N* is the number of isochromatic fringes, *f*_*σ*_ is fringe value, and *h* is the thickness of specimen.

**Figure 8:**
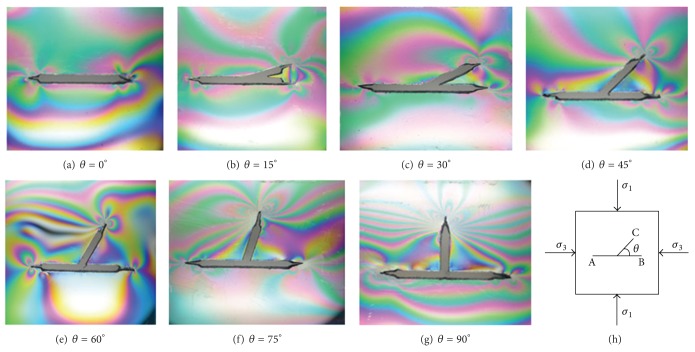
Isochromatic fringes of Y-shaped cracks under biaxial compression.

Figure 8 shows the test results of isochromatic fringes for the specimens with different branch angles *θ*. It can be seen that as *θ* increases, the fringes number near crack tips increases gradually, which means that crack SIF values increase, and as the branch angle is in the range between 60° and 75°, the fringes number is more. This means that the compressive strength is low, which can be proved from the test results of the compressive strength shown in Figure 4.

## 5. Conclusion

In order to study the fracture property of Y-shaped cracks under compression, compression tests by using a true triaxial loading device and the corresponding photoelastic experiments have been conducted. The stress intensity factors of Y-shaped cracks have been calculated by using ABAQUS code. In the numerical study, J-integration method has been applied in calculating stress intensity factors. Through the experimental and numerical studies, the following conclusions can be obtained.

1. For a Y-shaped crack under compression, at tips A and B, the mode I SIFs are always negative which will not induce fracturing, and their maximum mode II SIFs are much less than those at tip C.
2. As the branch angle is larger than 65°, the mode I SIF at tip C is positive, and as the branch angle is 50°, tip C has the largest *K*_II_ value.
3. The critical stress changes with the branch angles, and as the branch angle increases from 15° to 75°, the critical stress decreases, and as the branch angle is 30°, the principle stress is the largest, and as it is 75°, the principle stress is the lowest.

## Figures and Tables

**Table 1:** Calculation results of SIF (MPa∗m^1/2^) from ABAQUS code.

Angle	Crack A		Crack B		Crack C	
	*K*_I_	*K*_II_	*K*_I_	*K*_II_	*K*_I_	*K*_II_
15°	−0.270	−0.002	−0.22	−0.018	−0.168	−0.010
30°	−0.271	−0.008	−0.247	0.001	−0.124	−0.051
45°	−0.272	−0.017	−0.265	0.017	−0.067	−0.068
60°	−0.273	−0.026	−0.275	0.029	−0.013	−0.067
75°	−0.276	−0.034	−0.279	0.036	0.026	−0.039
90°	−0.279	−0.038	−0.279	0.038	0.041	0
